# Aging and Cognitive Health in Nepal: Prevalence of Dementia, Disability, and Functional Decline among the community residing people (aged≥60) in Nepal

**DOI:** 10.1002/alz70860_107544

**Published:** 2025-12-23

**Authors:** Prekshya Thapa, Stella‐Maria Paddick, Nidesh Sapkota, Ammu Lukose

**Affiliations:** ^1^ B.P. Koirala Institute of Health Sciences, Dharan, Kosi, Nepal; ^2^ Newcastle University, Newcastle Upon Tyne, United Kingdom; ^3^ Patan Academy of Health Sciences, Lalitpur, Lalitpur, Bagmati, Nepal; ^4^ Center for Brain Research (CBR), Indian Institute of Sciences (IISC), Bangalore, India

## Abstract

**Background:**

The much‐needed studies on local epidemiological and psychosocial burden of dementia and disability among elderly is lacking in Nepal which are needed for planning and policy making. Thus, this community‐based study was conducted to screen Dementia, Functional impairment and Disability among community residing elderly people.

**Method:**

This was a one‐stage house to house cross‐sectional epidemiological survey of all individuals aged ≥60 years in two wards of Dharan Sub Metropolitan City, Nepal. Measures used were Informant Questionnaire on Cognitive Decline (IQCODE) and Brief Community Screening Instrument for dementia (CSI‐D), Everyday Ability Scale (EASI) and Barthel Index. Data were collected by trained nurses from December 2021 to March 2022 using Kobo toolbox software and analyzed using SPSS‐16. Ethical clearance was obtained from the Nepal Health Research Council.

**Result:**

A total of 1009 (588 females) were enrolled in the study. Dementia was prevalent in 8.8% and 10.7% by Brief‐CSID and IQ‐CODE respectively. The bivariate analysis showed dementia was more frequent in ≥75 age group (OR 2.8[95% CI 1.9‐ 4.5]; *p* <0.001), having physical illness (OR 2.3[95%CI 1.3‐3.9]; *p* = 0.003), having hearing impairment (OR 1.9[95%CI 1.2‐3.0]; *p* = 0.004), and visual impairment (OR 1.9[95%CI 1.0‐3.2]; *p* = 0.023). Multivariate logistic regression analysis revealed dementia by Brief CSID was associated with ≥75 age group (AOR 2.296[1.418‐3.719], *p* = 0.001) and people using some form of mobility aids (AOR=2.022[1.19‐3.435], *p* = 0.009). Similarly, dementia by IQCODE was associated with illiterate groups (*p* = 0.02) and homemaker by occupation (*p* = 0.02). Functional impairment (EASI score ≥5) was present in 4.9%, and 19.5% had moderate disability by Barthel index.

**Conclusion:**

The study showed the prevalence of dementia among people aged ≥60 years old is high and associated with the number of risk factors. Physical illness and sensory impairments may be a modifiable risk factor in this population.

